# Enhancing Radiation Therapy Response in Prostate Cancer Through Metabolic Modulation by Mito-Lonidamine: A ^1^H and ^31^P Magnetic Resonance Spectroscopy Study

**DOI:** 10.3390/ijms26020509

**Published:** 2025-01-09

**Authors:** Stepan Orlovskiy, Pradeep Kumar Gupta, Fernando Arias-Mendoza, Dinesh Kumar Singh, Skyler Nova, David S. Nelson, Vivek Narayan, Cameron J. Koch, Micael Hardy, Ming You, Balaraman Kalyanaraman, Kavindra Nath

**Affiliations:** 1 Department of Radiology, University of Pennsylvania, Philadelphia, PA 19104, USA; stepano@sas.upenn.edu (S.O.); pradeep.gupta@pennmedicine.upenn.edu (P.K.G.); fernando.arias-mendoza@pennmedicine.upenn.edu (F.A.-M.); dinesh.singh@pennmedicine.upenn.edu (D.K.S.); snova@sas.upenn.edu (S.N.); dsnelson@pennmedicine.upenn.edu (D.S.N.); 2 Advanced Imaging Research Inc., Cleveland, OH 44114, USA; 3 Department of Medicine, University of Pennsylvania, Philadelphia, PA 19104, USA; vnarayan@pennmedicine.upenn.edu; 4 Department of Radiation Oncology, University of Pennsylvania, Philadelphia, PA 19104, USA; kochc@pennmedicine.upenn.edu; 5 Département de Chimie, Aix-Marseille University, CNRS, ICR, UMR 7273, 13013 Marseille, France; micael.hardy@univ-amu.fr; 6 Center for Cancer Prevention, Houston Methodist Cancer Center, Houston Methodist Research Institute, Houston, TX 77030, USA; myou@houstonmethodist.org; 7 Department of Biophysics, Medical College of Wisconsin, Milwaukee, WI 53226, USA; balarama@mcw.edu

**Keywords:** prostate cancer, magnetic resonance imaging, magnetic resonance spectroscopy, metabolic modulation, radiation therapy, lonidamine, mito-lonidamine

## Abstract

Radiation therapy (RT) is the cornerstone treatment for prostate cancer; however, it frequently induces gastrointestinal and genitourinary toxicities that substantially diminish the patients’ quality of life. While many individuals experience transient side effects, a subset endures persistent, long-term complications. A promising strategy to mitigate these toxicities involves enhancing tumor radiosensitivity, potentially allowing for lower radiation doses. In this context, mito-lonidamine (Mito-LND), an antineoplastic agent targeting the mitochondrial electron transport chain’s complexes I and II, emerges as a potential radiosensitizer. This study investigated Mito-LND’s capacity to augment RT efficacy and reduce adverse effects through comprehensive in vitro and in vivo assessments using hormone-sensitive and hormone-refractory prostate cancer models. Employing a Seahorse analysis and ^1^H/^31^P magnetic resonance spectroscopy (MRS), we observed that Mito-LND selectively suppressed lactate production, decreased intracellular pH, and reduced bioenergetics and oxygen consumption levels within tumor cells. These findings suggest that Mito-LND remodels the tumor microenvironment by inducing acidification, metabolic de-energization, and enhanced oxygenation, thereby sensitizing tumors to RT. Our results underscore the potential of Mito-LND as a therapeutic adjunct in RT to improve patient outcomes and reduce radiation-associated toxicities in early-stage prostate cancer.

## 1. Introduction

Prostate cancer is the second most common cancer in men worldwide, with 1.5 million new cases and 397,000 deaths having occurred in 2022 [1]. It is a leading cause of cancer death globally, ranking first in 52 countries and second only to lung cancer in the United States [1]. These statistics highlight the urgent need for novel treatment and prevention strategies. Prostate cancer is most diagnosed in men aged 45 to 74, with a median age of 67 [2]. Low-grade, localized cases generally have high survival rates with treatment or active surveillance, while high-grade metastatic cases have a 5-year survival rate of just 32% [3]. Prostate cancer can be suspected through a combination of clinical studies, including blood tests, magnetic resonance imaging, digital rectal exams, and routine health screenings [4]. Blood tests include the prostate-specific antigen (PCA) and the recent and more advanced prostate health index (PHI), among others [5]. However, the objective diagnosis of prostate cancer requires a biopsy, especially one that is image-guided, to improve the chances of accurately sampling cancer tissue [6].

Surgery and/or radiation therapy (RT) are the primary treatments for early-stage prostate cancer. As a foundational modality in cancer management, radiation therapy plays a critical role in treating early-stage prostate cancer. However, its therapeutic efficacy is frequently associated with collateral damage to surrounding healthy tissues, particularly within the gastrointestinal (GI) and genitourinary (GU) systems. Although advancements in precision delivery methods, such as intensity-modulated radiation therapy (IMRT) and proton therapy, have decreased the prevalence and severity of these adverse effects, the risk to adjacent normal tissues persists [7].

Microenvironment modulation, especially in metabolomics, could enhance the effects of RT. Notably, cancer cells often rely on altered metabolic processes supporting their rapid growth [8,9,10,11,12,13,14,15,16,17,18]. By selectively disrupting these pathways, metabolic modulators can increase tumor cell sensitivity to RT, enhancing its effectiveness and reducing doses. Our group has shown that tumor metabolism modulation is a promising approach in regard to reducing treatment-related toxicities, targeting metabolic pathways that are essential for cancer cell survival and proliferation [19]. This strategy can improve therapeutic efficacy and the sparing of healthy tissues, ultimately reducing the incidence of radiation-induced toxicities [19].

We have characterized and used lonidamine (LND) as a metabolic modulator that alters glycolysis in several types of cancers without disrupting the metabolism of healthy tissues [20]. Specifically, we previously reported that LND sensitizes the androgen-independent PC3 prostate cancer cell line to RT [19]. In the present work, we expand our observations to the androgen-dependent CWR22Rv1 prostate cancer cell line and compare the modulation effects of LND with mito-lonidamine (Mito-LND). Mito-LND is a modified version of lonidamine that is specifically designed to target mitochondria, making it significantly more potent and selective in targeting cancer cells by disrupting their mitochondrial function [19]. Mito-LND selectively inhibits the cancer mitochondrial electron transport chain’s complexes I and II and the oxygen consumption rate (OCR). It also decreases tumor hypoxia, enhancing oxygenation. Furthermore, Mito-LND has been shown to suppress lung tumor growth and brain metastasis by disrupting mitochondrial bioenergetics, promoting reactive oxygen species (ROS) production, oxidizing mitochondrial peroxiredoxin, inhibiting AKT/mTOR/p70S6K signaling, and triggering autophagic cell death in lung cancer cells. Notably, Mito-LND demonstrated no toxicity in mice, even when administered for eight weeks at doses 50 times higher than its effective cancer-inhibitory level. These findings highlight mitochondrial targeting of LND as a promising therapeutic strategy for exploring the role of autophagy in combating lung cancer, brain metastases, and other pathologies [21].

Our premise for the present work is that the increased effectiveness of Mito-LND, in comparison to LND, could allow for a more robust metabolic modulation, therefore enabling a more substantial reduction in RT dosage. This reduction may lower the risk of long-term side effects and improve patient outcomes. To evaluate this hypothesis, we conducted in vitro and in vivo studies using isolated cells and murine models of the PC3 and CWR22Rv1 human prostate cancer cell lines. We employed mitochondrial stress tests using Seahorse analyses, biochemical methods, and ^1^H and ^31^P magnetic resonance spectroscopy (MRS) to investigate the metabolic effects of LND and Mito-LND on these models and compare their impact when the models are treated with RT. Our results showed a significantly more potent and sustained tumor-selective reduction in lactate production, intracellular and extracellular pH, bioenergetics, and OCR with Mito-LND than was found with LND. These findings suggest that Mito-LND is a better choice for enhancing RT’s effectiveness in treating prostate cancer. By selectively targeting the energy machinery of the cancer cell, Mito-LND represents a novel strategy for enhancing the response to RT. Improving tumor sensitivity to RT could enable more effective treatment of patients with early-stage prostate cancer. It could also reduce the adverse effects of high-dose RT, ultimately leading to better patient outcomes and reducing morbidity, offering a promising future for prostate cancer treatment.

## 2. Results

### 2.1. Glucose and Lactate Measurements with LND and Mito-LND Treatment

**Figure 1:**
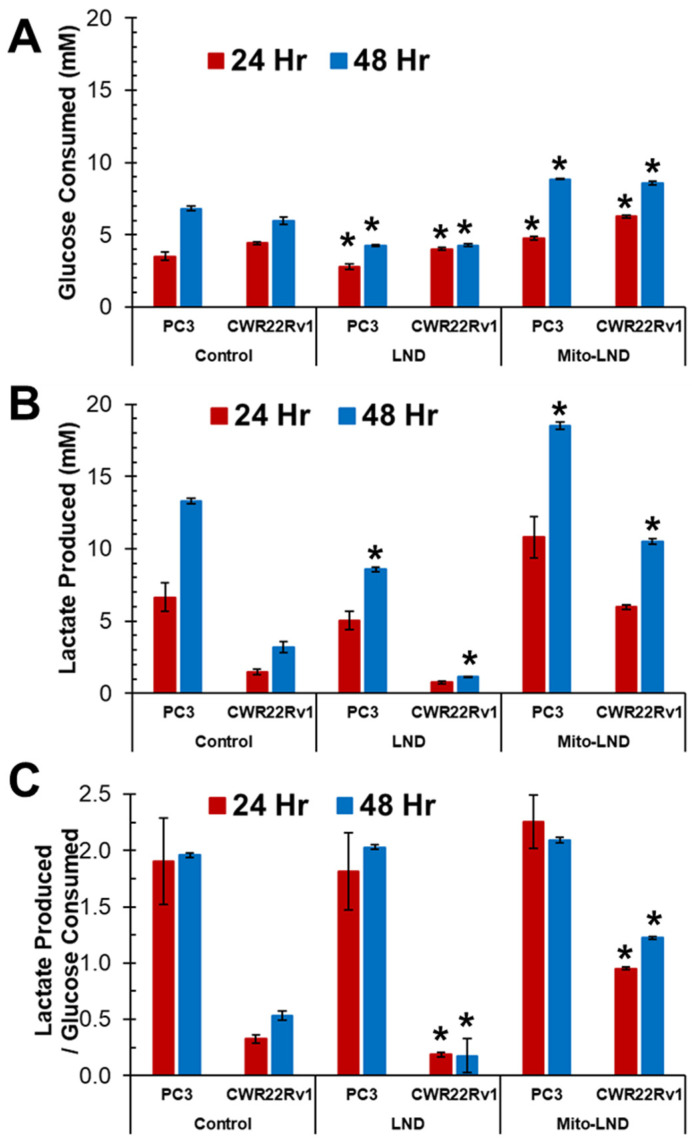
Glucose consumption and lactate production of PC3 and CWR22Rv1 prostate cancer cells under LND and Mito-LND treatment. PC3 and CWR22Rv1 cells were treated with either placebo (control), 200 µM LND, or 2 µM Mito-LND. Glucose (**A**), lactate (**B**), and the ratio of lactate produced/glucose consumed (**C**). Glucose and lactate were measured in the media at the 24- (red bars) and 48 h time points (blue bars) using a YSI 2300 STAT PLUS Biochemical Analyzer fitted with glucose or lactate membranes. Data in the figures are the net changes in the mean ± S.D. (*n =* 3). Asterisks indicate a statistically significant change (*p* < 0.01), comparing metabolite concentrations of either LND or Mito-LND vs. control using a Student’s *t*-test.

In Figure 1, we show the temporal glucose utilization (A) and lactate production (B) in PC3 and CWR22Rv1 cell lines with and without LND and Mito-LND treatments to understand their effect on cellular metabolism. We measured similar glucose and lactate concentrations in the basal media at the start of the experiment (i.e., pretreatment); for PC3, the values were 10.0 ± 0.08 and 1.66 ± 0.04 mM, respectively, and, for CWR22Rv1, they were 10.1 ± 0.1 and 1.63 ± 0.05 mM, respectively.

As expected, PC3, the hormone-treatment-refractory cell line, depleted glucose significantly faster than CWR22Rv1, the hormone-dependent cell line (67% vs. 59% glucose reduction by the end of 48 h, respectively; *p* = 0.001). Interestingly, LND decreased glucose utilization marginally in both cell types by 24 h and drastically by 48 h. However, Mito-LND had the opposite effect. Mito-LND increased glucose consumption and significantly depleted the extracellular media of glucose (about 90% depletion by 48 h).

As anticipated, glucose depletion in the extracellular media was mirrored by increased lactate production in both cell types while untreated, though the rates differed. By 48 h, the extracellular lactate production in PC3 was 8.9-fold compared to only 2.8-fold for CWR22Rv1. In LND-treated cells, lactate production slowed down significantly. Interestingly, though 90% of the glucose was depleted in both the cell types with Mito-LND treatment by 48 h, the lactate production was significantly different. In PC3 cells, approximately 20 mM of lactate (12-fold) was detected as compared to only 12 mM (7.6-fold) for CWR22Rv1 cells. To further characterize the metabolic phenotype of the models tested and the changes in that phenotype observed with the metabolic modulators, the ratio of lactate produced and the glucose consumed in the external medium was determined (C). These results show a highly glycolytic phenotype for PC3 with little to no change with in vitro incubation. Additionally, CWR22Rv1 showed a divergent effect, with LND having a lower ratio (lower ratio of lactate exported to glucose consumed) and Mito-LND demonstrating an increase in the ratio compared to basal conditions.

### 2.2. Seahorse Mitochondrial Stress Test Assay

**Figure 2:**
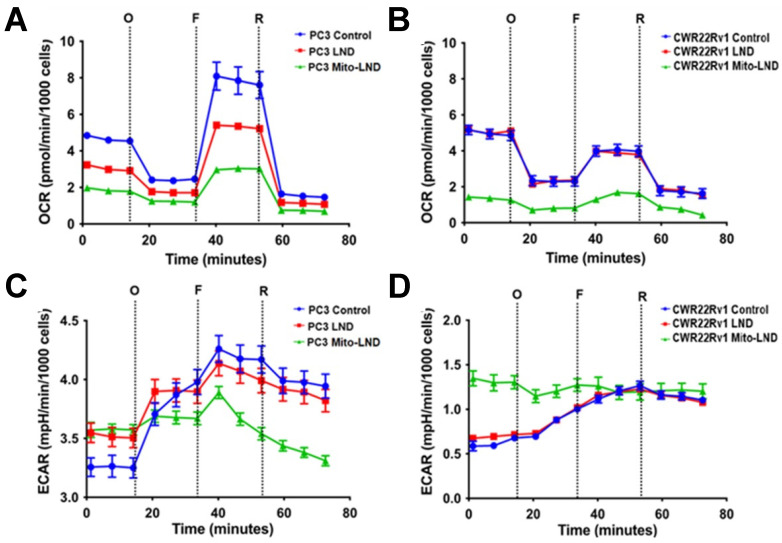
Seahorse mitochondrial stress test on PC3 and CWR22Rv1 (CWR) prostate cancer cells under LND and Mito-LND treatment. The figure shows the effect of 200 µM LND and 2 µM Mito-LND at 24 h on the mitochondrial function of PC3 (**A**,**C**) and CWR22Rv1 cells (**B**,**D**). The test was carried out using the following reagents: Oligomycin (O), FCCP (F), and Rotenone/Antimycin-A (R). Oxygen consumption rate (OCR) of PC3 (**A**) and CWR22Rv1 cells (**B**). Extracellular acidification rate (ECAR) of PC3 (**C**) and CWR22Rv1 cells (**D**). Data were gathered on a Seahorse XFe96 Extracellular Flux Analyzer and are shown as mean ± SD (*n* = 14).

Treatment with 2 µM Mito-LND for 24 h significantly decreased the OCR of PC3 and CWR22Rv1 cells relative to control (Figure 2). This relatively low concentration of Mito-LND had a more significant effect on the OCR than 200 µM LND on PC3 and CWR22Rv1 cells at basal and stressed conditions. LND treatment decreased the OCR in PC3 cells but had no OCR effect in CWR22Rv1 cells. A similar trend was observed in the extracellular acidification rate (ECAR) of CWR22Rv1 cells, where there was no difference between the control and 200 µM LND treatment groups. In contrast, Mito-LND treatment significantly increased the ECAR of CWR22Rv1 cells, and the cells remained at an elevated ECAR throughout most of the experiment. In comparison, PC3 cells treated with Mito-LND experienced a significant decrease in their ECAR at around 40 min into the experiment. LND treatment significantly increased the ECAR of PC3 cells at basal conditions, matching the Mito-LND, but it was not significant compared to the control for the remainder of the study.

### 2.3. Seahorse Cell Energy Phenotype

**Figure 3:**
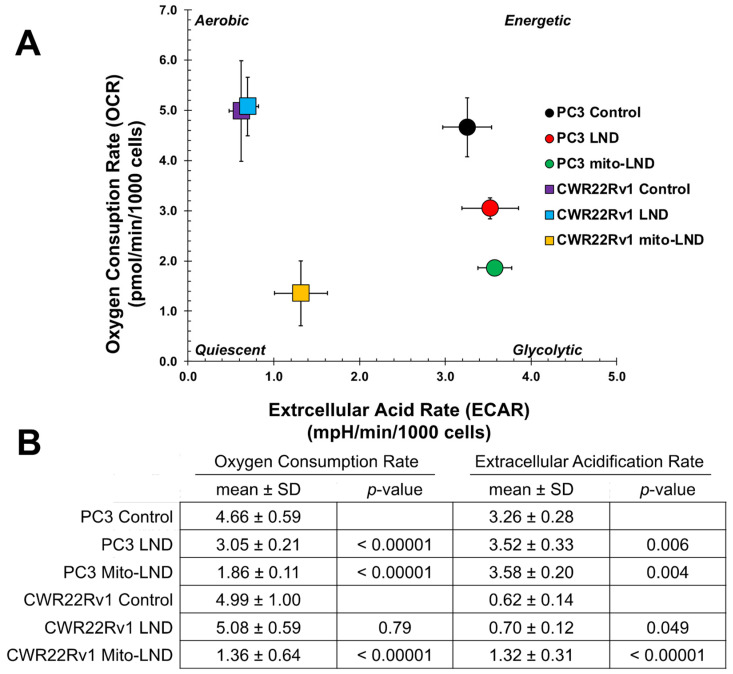
Effect of 200 µM LND and 2 µM Mito-LND on the cell energy phenotypes of PC3 and CWR22Rv1 after 24 h of treatment. (**A**) Seahorse energy phenotype map plotting the basal oxygen consumption rates (OCRs) and basal extracellular acidification rates (ECARs) in the control, LND, and Mito-LND groups shown in Figure 2. (**B**) Quantified OCR and ECAR values of cells with standard deviation and *p*-values comparing controls to treated groups. Data were gathered via a Mito Stress Test Assay using the Seahorse XFe96 Extracellular Flux Analyzer and shown as mean ± SD (*n* = 14).

As shown in Figure 3A, PC3 and CWR22Rv1 cells show similar values for their basal OCR (control), while PC3 cells have a significantly higher ECAR at baseline than CWR22Rv1 cells (*p* < 0.001). These data demonstrate that PC3 cells at baseline have a more energetic cell phenotype that relies on oxidative phosphorylation and glycolysis in comparison to CWR22Rv1 cells, which have a predominantly aerobic phenotype that relies heavily on oxidative phosphorylation but with lower levels of glycolysis for energy production. Figure 3 also shows that LND produces a significant decrease in the basal OCR of PC3 cells (34.5%) and a significant increase in the ECAR. LND did not significantly change the OCR or ECAR in CWR22Rv1 cells. Conversely, significant decreases from the basal OCR and significant increases in the ECAR in both cell lines were recorded with Mito-LND. Given the ECAR differences in the cell lines at baseline, the OCR and ECAR changes after LND in PC3 and Mito-LND in both cell lines produce different metabolic phenotypes in the two prostate cancer cell lines. While PC3 became more glycolytic with both drugs, CWR22Rv1 did not change its aerobic phenotype with LND but became more quiescent with Mito-LND.

### 2.4. Prostate Cancer Cell Clonogenic Assay

**Figure 4:**
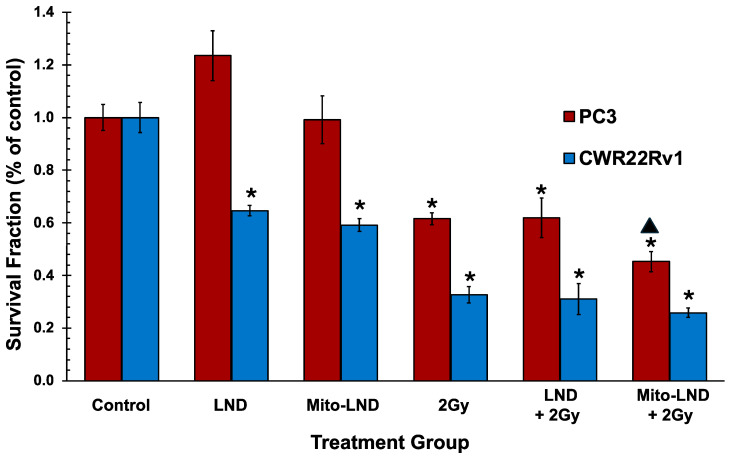
Proliferative capacity presented as a survival fraction of PC3 and CWR22Rv1 with LND, Mito-LND, and radiation. Clonogenic assay showing the survival fraction of PC3 and CWR22Rv1 prostate cancer cells treated with 200 µM LND, 2 µM Mito-LND, and 2 Gy radiation (RT). Each treatment was given alone or through combining RT with LND or Mito-LND. The experiment was performed with *n =* 4 for CTRL, RT alone, LND + RT, and Mito-LND + RT groups, and *n* = 8 for LND alone and Mito-LND alone groups. The data are represented as mean ± SEM. The asterisks indicate a statistical significance of *p* < 0.01 relative to the control group, while the black triangle indicates a significance of *p* < 0.05 relative to the RT group.

The proliferative capacity of PC3 and CWR22Rv1 prostate cancer cells was tested using clonogenic assays. As shown in Figure 4, in these studies, 200 µM LND alone or 2 µM Mito-LND alone produced no significant changes in the proliferative capacity in PC3 cells but significantly decreased proliferation in CWR22Rv1. In comparison, 2 Gy of RT produced the expected significant reduction in proliferative capacity in both cell lines. Figure 4 also shows that LND, combined with RT, did not significantly affect the proliferative capacity of both cell lines relative to radiation alone. However, the Mito-LND/RT combination significantly enhanced the effect of RT on the proliferative capacity of androgen-independent PC3 prostate cancer cells. A slight proliferation decrease was also recorded for this combination in the androgen-dependent CWR22Rv1, but it was insignificant.

### 2.5. In Vivo ^1^H MRS

**Figure 5:**
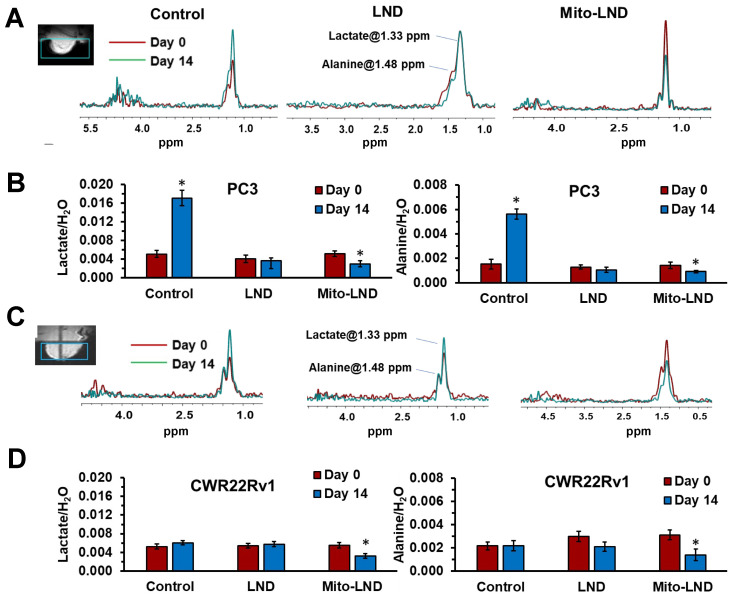
Lactate and alanine assessment by in vivo ^1^H MRS. (**A**,**C**) show representative ^1^H MRS spectra localized in tumors of PC3 and CWR22Rv1 cells in mice xenografts, respectively. They were acquired using the HDMD-Sel-MQC pulse sequence. The area integration of the lactate and alanine spectral signals, normalized to the water signal, are shown in (**B**) for PC3 and in (**D**) for CWR22Rv1 xenografts. These measurements were carried out in controls (vehicle) and animals treated with LND or Mito-LND at an oral dose of 7.5 µmol/kg once daily for 14 days. An asterisk (*) indicates a statistical significance (*p* < 0.05) when comparing the control (*n* = 5) and treated groups (*n* = 5) on Day 14.

Data from in vivo ^1^H MRS exams of PC3 (Figure 5A,B) and CWR22Rv1 prostate cancer xenografts (Figure 5C,D) were obtained on Day 0 and Day 14 of treatment. These MRS exams utilized a selective pulse sequence for visualizing lactate and alanine (see Section 4). While Figure 5A,C show representative spectra of these studies, Figure 5B,D show the result of integrating the spectral peak areas of lactate and alanine normalized to the water signal in the tumor volume selected (green square overlaying the images in the insets of Figure 5). No significant differences in lactate and alanine metabolites were observed between the PC3 and CWR22Rv1 models on Day 0. However, by Day 14, a significant increase in lactate and alanine levels was evident in the PC3 compared to the CWR22Rv1 within the control groups, indicating differential metabolic responses over time.

Figure 5 also shows that LND in PC3 cells prevented the increase in both metabolites seen in the control group on Day 14. However, LND did not change the lactate or alanine content in CWR22Rv1 cells on Day 14. In contrast, Mito-LND reduced the lactate and alanine content in PC3 cells on Day 14 compared to their value in the control group (Day 0). Mito-LND significantly reduced both metabolites on Day 14 compared to the control group on Day 0. These significant reductions in metabolites on Day 14 were also demonstrated in CWR22Rv1 cells treated with Mito-LND.

### 2.6. In Vivo ^31^P MRS

**Figure 6:**
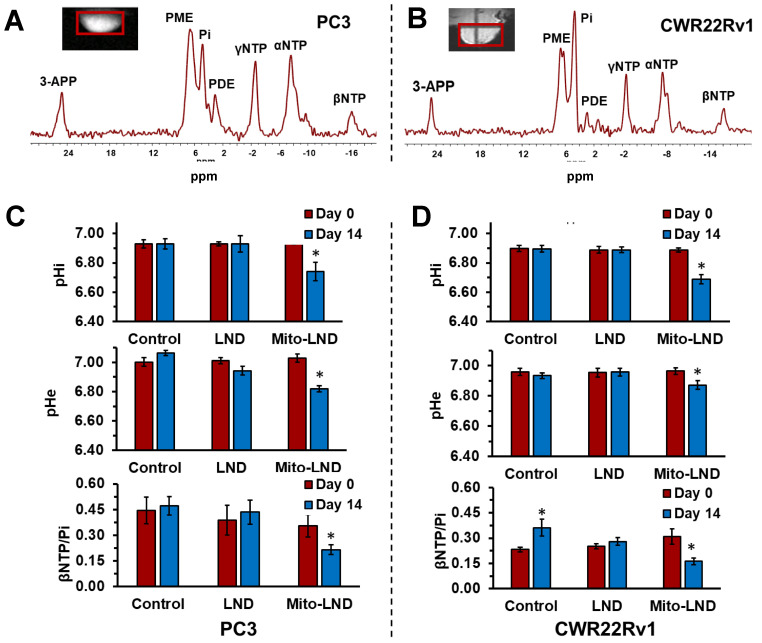
In vivo measurement of intracellular (pHi), extracellular pH (pHe), and bioenergetics (βNTP/Pi) by ^31^P MRS. At the top, representative in vivo localized ^31^P MRS spectra of PC3 (**A**) and CWR22Rv1 (**B**) prostate cancer xenografts are shown. Below are the measurements of pHi, pHe, and βNTP/Pi measured by the analysis of ^31^P MRS in untreated (controls) and LND- and Mito-LND-treated PC3 (**C**) and CWR22Rv1 xenografts (**D**). LND and Mito-LND were administered orally at a dose of 57.5 µmole/kg once daily for 14 days. Peak assignments: 3-APP, 3-aminopropylphosphonat; PME, phosphomonoesters; Pi, inorganic phosphate; PDE, phosphodiesters; γ-, α-, and β-NTP, the γ-, α-, and β-moieties of nucleoside triphosphates. An asterisk (*) indicates a statistical significance (*p* < 0.05) between the control group (*n* = 5; red bars) and the treated group (*n* = 5; green bars) during the follow-up period.

Figure 6 shows in vivo ^31^P MRS data obtained from tumors of PC3 and CWR22Rv1 prostate cancer cells xenografted in mice. Spectra were localized to the tumors using a single voxel technique, as shown in the MR images (insets of Figure 6). In these images, a red rectangle in red is the projection of the selected voxel. Representative ^31^P MR tumor spectra acquired from PC3 and CWR22Rv1 prostate cancer xenografts are shown in Figure 6A,B (top), respectively.

As the spectra of Figure 6 show, each of the three phosphorus moieties of nucleoside triphosphates (NTPs) gives a distinctive spectral signal. To determine the energetic status in the tumor, we integrated the βNTP and Pi signals to obtain the βNTP/Pi ratio. Although all β-phosphates of the NTPs are included in the βNTP peak, we consider this signal a better measure of bioenergetics, as all NTPs are included, their pools are at equilibrium, and the adenosine derivative (ATP) is the most concentrated by several orders of magnitude. We selected the β-resonance of NTP, as it is apart from the rest of the ^31^P signals and, hence, its integration is straightforward.

Below the spectra in Figure 6, the mean values of pHi, pHe, and βNTP/Pi in PC3 (A) and CWR22Rv1 (B) of untreated (control) and LND- and Mito-LND-treated xenografts obtained at Day 0 and Day 14 are shown. The pHi and pHe measurements are unremarkable in both xenografts’ control and LND groups. However, Mito-LND significantly reduced both pH values at Day 12 of treatment in both xenografts. Notably, the βNTP/Pi mean value is significantly higher in the control groups of PC3 in comparison to CWR22Rv1. Furthermore, on Day 12 in the control group of CWR22Rv1, the βNTP/Pi increased significantly from its value on Day 0. While LND did not affect the βNTP/Pi value in PC3 xenografts, it prevented its increase, as shown in the control group on Day 12. In contrast, Mito-LND significantly reduces the βNTP/Pi values in both xenografts below the level of its control group on Day 12.

## 3. Discussion

Prostate cancer cells, like many other cancers, exhibit metabolic reprogramming. They often rely on enhanced glycolysis (the “Warburg effect”) for energy production and survival, including the active production of citrate, which is typically sustained by glycolysis in the prostate [22]. As shown here, Mito-LND, a mitochondria-targeted analog of LND, is a novel modulator that disrupts these metabolic processes in prostate cancer models, offering a promising avenue for future treatment [21,23]. Mito-LND inhibits mitochondrial complexes I and II, reducing ATP production and disrupting oxidative phosphorylation, likely leading to increased oxygenation and ROS induction by RT [21]. This accumulation of ROS exacerbates DNA damage in cancer cells when combined with RT, enhancing radiosensitivity. In hormone-dependent prostate cancer, Mito-LND-induced metabolic modulation might have additional potential by interfering with androgen receptor signaling, which plays a crucial role in regulating cancer metabolism. Disrupting androgen receptor-regulated metabolic pathways further sensitizes prostate cancer cells to radiation-induced DNA damage [24]. This dual targeting of mitochondrial metabolism and androgen receptor signaling offers a potent approach to enhancing the efficacy of RT in hormone-sensitive settings. In hormone-independent CRPC settings, prostate cancer cells rely on alternative metabolic pathways, such as oxidative phosphorylation, to survive [25]. Mito-LND stresses these cancer cells by targeting mitochondrial bioenergetics, making them more susceptible to RT.

RT is a critical treatment for both hormone-dependent (castration-sensitive) and hormone-independent (castration-resistant) prostate cancers, though clinical outcomes vary [26]. In hormone-dependent cases, RT combined with androgen deprivation therapy (ADT) improves progression-free and overall survival, especially in patients with high-risk or localized disease. This combination is the standard of care, showing significant benefits for controlling tumor growth by suppressing androgen-driven cancer proliferation [26,27]. For castration-resistant prostate cancer (CRPC), radiation remains valuable, particularly for localized treatments like bone metastases. However, these cancers are more aggressive and resistant to conventional therapies, making combination approaches necessary. One promising strategy to optimize RT therapy is metabolic modulation, which targets the altered energy metabolism in prostate cancer [26,28]. In the present study, LND and Mito-LND function as metabolic modulators. Still, Mito-LND demonstrated a more substantial radiosensitizer effect, as shown in Figure 4. Therefore, Mito-LND has the potential to significantly improve treatment efficacy by enhancing RT response in prostate cancer, especially in hormone-independent settings.

In our in vitro studies (Figure 1, Figure 2 and Figure 3), we observed that Mito-LND caused rapid glucose depletion from the media with a concomitant increase in lactate extrusion (Figure 1) and reduced OCRs (Figure 2 and Figure 3), indicating rewiring of the cellular metabolism. These Mito-LND-related in vitro changes suggest increased tumor glycolysis with increased lactate extrusion and reduced bioenergetics. We corroborated this suggestion in our in vivo studies. Figure 5 shows that LND and Mito-LND prevent lactate and alanine intracellular accumulations in the untreated (control) group of the PC3 cell line on Day 14 (Figure 5B). Conversely, given that CWR22Rv1 does not increase lactate and alanine at Day 14 (Figure 5D), the LND effect is negligible in this cell line. However, Mito-LND prevented a tumor lactate increase in the PC3 cell line and significantly reduced it from the control values (Day 0) in both cell lines (Figure 5B,D). The intratumor reduction in lactate and alanine by Mito-LND correlates with the augmented lactate extrusion shown in Figure 1. The differences seen in the in vitro (Figure 1) and in vivo (Figure 5) systems are due to the varied complexities of the experimental protocols. The in vitro system is an integration of the changes observed over time in the extracellular media during the period of incubation, where the in vivo measurements are those at the time points (Day 0 and 14) indicated after the initiation of treatment in the animals that are subject to variation caused by both changes in tumor phenotype and tumor microenvironment. In other words, Figure 1 measures the lactate accumulation outside the cells, while Figure 5 measures lactate in the tumor, which is primarily inside the cells. Of note, it is interesting to speculate about the differences seen in the models studied in regard to their changing basal phenotypes in terms of growth. The PC3 model seems to show an increasing dependance on glycolysis indicated by higher levels over time in both lactate and alanine, whereas, in the CWR22Rv1, there is no statistically significant change in these ^1^H detectable metabolites. Additionally, Figure 6 depicts increased intra- and extracellular acidification, the last being due to the increased lactate extrusion. The figure also objectively demonstrates that the diminished OCR caused by Mito-LND (Figure 2 and Figure 3) correlates with the reduction in the energy status in the cell in vivo (i.e., significant reductions in βNTP/Pi in Figure 6). As the intracellular increase in acidification in Figure 6 does not match the intracellular lactate levels in Figure 5, we believe that the cancer cell affected by Mito-LND actively generates other organic acids besides lactate to compensate for its increased extrusion and survival. These results underscore Mito-LND’s ability to disrupt tumor metabolism, making it an attractive radiosensitizer, especially for aggressive, hormone-independent cancers.

Another critical challenge in RT is tumor hypoxia, which limits its effectiveness because oxygen is required for ROS formation. Hypoxic regions in tumors are often resistant to radiation [29]. Mitochondria-targeted drugs (e.g., Mito-metformin, atovaquone, Mito-atovaquone, IACS-010759) have been shown to inhibit mitochondrial respiration, decrease tumor hypoxia, increase tumor oxygenation, inhibit hypoxic gene expression, and increase radiosensitivity in tumor cells and cancer patients [30,31,32,33,34,35,36]. This study shows Mito-LND’s ability to disrupt glycolysis and oxidative phosphorylation, enhancing tumor oxygenation and tumor radiosensitivity. Although the glycolytic shift enhanced by Mito-LND is temporary, our results suggest that prolonged exposure to the drug would not only deplete glucose as the nutrient source (Figure 1) but also lead to oxidative stress, favoring cell death (Figure 4). Furthermore, in altering the tumor microenvironment, Mito-LND, as evidenced by our in vivo results, could reduce immune evasion and enhance anti-tumor immune responses, making Mito-LND a valuable adjunct in multi-modal cancer therapy [37,38].

Briefly, Mito-LND has advantages in comparison to other radiosensitizers and metabolic modulators that typically enhance radiation-induced DNA damage or impair DNA repair mechanisms. Examples include hypoxic cell radiosensitizers like nimorazole or agents targeting specific pathways (e.g., PARP inhibitors) [39,40]. Mito-LND offers less toxicity compared to altering cancer cell metabolism, such as glycolysis inhibitors (e.g., 2-deoxyglucose) or drugs targeting mitochondrial function (e.g., metformin, which disrupts complex I) [41,42,43]. However, Mito-LND’s dual role in disrupting mitochondrial function and inducing autophagic cell death seems to be better tolerated and has a broader anti-tumor effect compared to agents that focus solely on enhancing DNA damage or metabolic interference [21].

Our preclinical studies have demonstrated that Mito-LND effectively radiosensitizes various cancer cell lines, including prostate cancer. By targeting both glycolytic and mitochondrial pathways, Mito-LND enhances the cytotoxic effects of radiation while sparing normal tissues. This selective action makes Mito-LND a promising therapeutic option for overcoming radiation resistance in hormone-independent prostate cancers.

## 4. Materials and Methods

### 4.1. Prostate Cell Cultures

Androgen-independent PC3 (Accession CVCL_0035) and androgen-dependent CWR22Rv1 (Accession CVCL_1045) prostate cancer cells were obtained from ATCC (Manassas, VA, USA). The cells were cultured in RPMI 1640 medium (Invitrogen Corporation, Carlsbad, CA, USA)supplemented with 10% fetal bovine serum (Cytiva–HyClone, Logan, UT, USA), 1% sodium pyruvate, and 1% penicillin–streptomycin (*V*/*V*, 100 U/mL final)(Invitrogen Corporation, Carlsbad, CA, USA). Cells were grown in T-75 or T-182 tissue culture flasks (Genesee Scientific, El Cajon, CA, USA) and held in an incubator at 5% CO_2_ and 37 °C until the desired confluency. When the cells were around 90% confluent, they were washed with Hanks Balanced Salt Solution (Cell Center Service Facility, Philadelphia, PA, USA) and incubated in cold 0.05% trypsin-EDTA (Invitrogen Corporation, Carlsbad, CA, USA) for 10 min before being split (1:16) into new flasks. Cells were counted with a hemocytometer and trypan blue stain (Mediatech Inc., Herndon, VA, USA) when specific cell numbers were needed.

### 4.2. Seahorse Mito Stress Test Assays

An XFe96 Seahorse Extracellular Flux Analyzer (Agilent Technologies; Santa Clara, CA, USA) was used to measure the OCR and ECAR of prostate cancer cells in vitro via the Mito Stress Test Assay. The assay media was formulated to match the culture media by supplementing Seahorse XF Base Medium with 5 mM glucose, 2 mM L-glutamine, and 1 mM sodium pyruvate. The Seahorse assay media was prepared at 7.4 pH for use in the instrument, as specified in the Mito Stress Test Assay Guide. PC3 and CWR22Rv1 prostate cancer cells were treated with 200 µM LND (Santa Cruz Biotechnology, Inc. Dallas, TX, USA) and 2 µM Mito-LND (Medical College of Wisconsin, Milwaukee, WI, USA) for 24 h prior to the experiment. During the experiment, 2 × 10^4^ PC3 or CWR22Rv1 cells were seeded, per well, into an XF96 cell culture microplate and a Mito Stress Test Assay was conducted.

### 4.3. Glucose Lactate Measurements with YSI 2300 STAT Plus

Glucose consumption and lactate production rates of PC3 and CWR22Rv1 cells were determined using a YSI 2300 STAT Plus biochemistry analyzer (YSI, Inc., Yellow Springs, OH, USA) fitted with glucose and lactate membranes and calibrated with a YSI 2747 calibration solution. PC3 and CWR22Rv1 cells were seeded in T-75 flasks at a density of 2 × 10^6^ cells in 20 mL of supplemented RPMI 1640 media containing 200 μM LND or 2 μM Mito-LND. Extracellular glucose and lactate concentrations were measured 24 and 48 h after the start of treatment.

### 4.4. Human Prostate Cancer Xenografts in Athymic Nude Mice

PC3 and CWR22Rv1 prostate cancer xenografts were grown in athymic nude mice. Briefly, 7 × 10^6^ cells in 100 µL Hanks Balanced Salt Solution were inoculated subcutaneously into the right flank of nude mice. The xenograft doubling times were calculated by measuring tumor growth in untreated mice with a caliper using the formula for the volume of a half-ellipsoid, *V* = (π/6) (*l* × *w* × *d*), where *l*, *w*, and *d* are the tumor’s length, width, and depth, respectively.

### 4.5. Non-Invasive Magnetic Resonance Spectroscopy (MRS) Measurements

^1^H and ^31^P MRS were performed on a 9.4 T/31 cm horizontal bore Bruker spectrometer (Bruker Corp., Billerica, MA, USA) after positioning the subcutaneous tumor in a dual-frequency slotted-tube resonator. The pHi, pHe, βNTP/Pi, lactate, and alanine parameters were measured immediately before (Day 0) and on Day 14 after starting administration of the vehicle (control group), LND, or Mito-LND (*n* = 5 per treatment group). The dose for both drugs was 7.5 µmol/kg taken orally, once daily, for 14 days. Physiological monitoring was maintained during the whole experiment. Data acquisition, post-processing, and parameter estimation procedures were performed as described elsewhere [19,20,44,45,46,47,48].

### 4.6. Clonogenic Assay Radiation Experiments

PC3 and CWR22Rv1 cells were grown in T-75 tissue culture flasks in RPMI 1640 medium supplemented with 10% fetal bovine serum, 1% sodium pyruvate, and 1% penicillin–streptomycin (*V*/*V*, 100 U/mL final) at 5% CO_2_ and 37 °C until desired confluency (~5 × 10^6^ cells). A Cs-137 irradiator (J L Shepherd & Assoc., Mark 1, San Fernando, CA, USA) was used to treat PC3 prostate cancer cells at various radiation doses (1–4 Gy). A clonogenic assay was then conducted to determine the optimal RT dose in a combination experiment with LND and Mito-LND therapy. Based on this clonogenic dose–response curve, a 2 Gy dose was selected due to its sufficient dynamic range to demonstrate increased response with LND and Mito-LND. PC3 and CWR22Rv1 cells were treated with 200 µM LND and 2 µM Mito-LND for 40 min to achieve significant acidification and de-energization during the clonogenic assay radiation experiments. The treated cells were then given a 2 Gy dose of RT using the irradiator. The cells were then incubated for 1 h, trypsinized, and seeded in 10 cm tissue culture plates. One hundred cells were seeded for the control group, while 100, 200, 400, 800, and 1000 cells were seeded for LND, Mito-LND, LND + 2 Gy, and Mito-LND + 2 Gy groups. A colony was defined as 50 or more cells, and each colony was counted using a microscope to confirm viable colonies.

### 4.7. Statistical Analysis

Student’s *t*-tests assuming homogeneity of variances were performed in all data using SPSS 20 (IBM Corp., Armonk, NY, USA), considering significance at σ = 0.05.

## 5. Conclusions

Our promising strategy to optimize RT therapy in prostate cancer is microenvironment modulation, targeting cancer’s altered energy metabolism. The present study, which employed robust in vitro and in vivo experimental methods, tested the metabolic effects of LND and Mito-LND in prostate cancer models, as both drugs are recognized metabolic modulators. Mito-LND demonstrated a more substantial radiosensitizer effect than LND in the models. This effect highlights Mito-LND’s ability to disrupt prostate cancer’s glycolysis and oxidative phosphorylation, affecting its oxygenation and oxidative stress and favoring tumor cell death.

By altering the tumor microenvironment, Mito-LND could reduce immune evasion and enhance anti-tumor immune responses, making it a valuable adjunct in multi-modal cancer therapy. Mito-LND could also interfere with androgen receptor signaling, which is crucial in regulating metabolism in both healthy and malignant prostate tissues. This dual targeting of mitochondrial metabolism and androgen receptor signaling by Mito-LND strengthens its potential to enhance the efficacy of RT in prostate cancer. Our findings confirm that Mito-LND can effectively target metabolism to improve prostate cancer treatment. However, the need for more research to fully elucidate the potential of Mito-LND in enhancing cancer therapy in clinical settings is crucial. The present study paves the way for human studies, as our in vivo MRS exams are fully translatable to the clinical realm, instilling hope for the potential use of Mito-LND in cancer therapy.

## Data Availability

All data are available upon request.

## References

[1] Bray F., Laversanne M., Sung H., Ferlay J., Siegel R.L., Soerjomataram I., Jemal A. (2024). Global cancer statistics 2022: GLOBOCAN estimates of incidence and mortality worldwide for 36 cancers in 185 countries. CA Cancer J. Clin..

[2] National Cancer Institute: SEER Program Cancer Stat Facts: Prostate Cancer. https://seer.cancer.gov/statfacts/html/prost.html.

[3] Fairey A., Paproski R.J., Pink D., Sosnowski D.L., Vasquez C., Donnelly B., Hyndman E., Aprikian A., Kinnaird A., Beatty P.H. (2023). Clinical analysis of EV-Fingerprint to predict grade group 3 and above prostate cancer and avoid prostate biopsy. Cancer Med..

[4] Sekhoacha M., Riet K., Motloung P., Gumenku L., Adegoke A., Mashele S. (2022). Prostate Cancer Review: Genetics, Diagnosis, Treatment Options, and Alternative Approaches. Molecules.

[5] Agbetuyi-Tayo P., Gbadebo M., Rotimi O.A., Rotimi S.O. (2024). Advancements in Biomarkers of Prostate Cancer: A Review. Technol. Cancer Res. Treat..

[6] Watts K.L., Frechette L., Muller B., Ilinksy D., Kovac E., Sankin A., Aboumohamed A. (2020). Systematic review and meta-analysis comparing cognitive vs. image-guided fusion prostate biopsy for the detection of prostate cancer. Urol. Oncol. Semin. Orig. Investig..

[7] Wisdom A.J., Yeap B.Y., Michalski J.M., Horick N.K., Zietman A.L., Christodouleas J.P., Kamran S.C., Parikh R.R., Vapiwala N., Mihalcik S. (2024). Setting the Stage: Feasibility and Baseline Characteristics in the PARTIQoL Trial Comparing Proton Therapy Versus Intensity Modulated Radiation Therapy for Localized Prostate Cancer. Int. J. Radiat. Oncol. Biol. Phys..

[8] Goodwin M.L., Gladden L.B., Nijsten M.W., Jones K.B. (2014). Lactate and cancer: Revisiting the warburg effect in an era of lactate shuttling. Front. Nutr..

[9] Koppenol W.H., Bounds P.L., Dang C.V. (2011). Otto Warburg’s contributions to current concepts of cancer metabolism. Nat. Rev. Cancer.

[10] Liberti M.V., Locasale J.W. (2016). The Warburg Effect: How Does it Benefit Cancer Cells?. Trends Biochem. Sci..

[11] Vander Heiden M.G., Cantley L.C., Thompson C.B. (2009). Understanding the Warburg effect: The metabolic requirements of cell proliferation. Science.

[12] Ahmad F., Cherukuri M.K., Choyke P.L. (2021). Metabolic reprogramming in prostate cancer. Br. J. Cancer.

[13] Cutruzzola F., Giardina G., Marani M., Macone A., Paiardini A., Rinaldo S., Paone A. (2017). Glucose Metabolism in the Progression of Prostate Cancer. Front. Physiol..

[14] Fidelito G., Watt M.J., Taylor R.A. (2021). Personalized Medicine for Prostate Cancer: Is Targeting Metabolism a Reality?. Front. Oncol..

[15] Pujana-Vaquerizo M., Bozal-Basterra L., Carracedo A. (2024). Metabolic adaptations in prostate cancer. Br. J. Cancer.

[16] Wanjari U.R., Mukherjee A.G., Gopalakrishnan A.V., Murali R., Dey A., Vellingiri B., Ganesan R. (2023). Role of Metabolism and Metabolic Pathways in Prostate Cancer. Metabolites.

[17] Ambrosini G., Cordani M., Zarrabi A., Alcon-Rodriguez S., Sainz R.M., Velasco G., Gonzalez-Menendez P., Dando I. (2024). Transcending frontiers in prostate cancer: The role of oncometabolites on epigenetic regulation, CSCs, and tumor microenvironment to identify new therapeutic strategies. Cell Commun. Signal..

[18] Ge R., Wang Z., Cheng L. (2022). Tumor microenvironment heterogeneity an important mediator of prostate cancer progression and therapeutic resistance. npj Precis. Oncol..

[19] Orlovskiy S., Gupta P.K., Roman J., Arias-Mendoza F., Nelson D.S., Koch C.J., Narayan V., Putt M.E., Nath K. (2024). Lonidamine Induced Selective Acidification and De-Energization of Prostate Cancer Xenografts: Enhanced Tumor Response to Radiation Therapy. Cancers.

[20] Nath K., Roman J., Nelson D.S., Guo L., Lee S.C., Orlovskiy S., Muriuki K., Heitjan D.F., Pickup S., Leeper D.B. (2018). Effect of Differences in Metabolic Activity of Melanoma Models on Response to Lonidamine plus Doxorubicin. Sci. Rep..

[21] Cheng G., Zhang Q., Pan J., Lee Y., Ouari O., Hardy M., Zielonka M., Myers C.R., Zielonka J., Weh K. (2019). Targeting lonidamine to mitochondria mitigates lung tumorigenesis and brain metastasis. Nat. Commun..

[22] Beier A.K., Puhr M., Stope M.B., Thomas C., Erb H.H.H. (2023). Metabolic changes during prostate cancer development and progression. J. Cancer Res. Clin. Oncol..

[23] Gupta P.K., Orlovskiy S., Roman J., Pickup S., Nelson D.S., Glickson J.D., Nath K. (2023). pH-dependent structural characteristics of lonidamine: ^1^H and ^13^C NMR study. RSC Adv..

[24] Dai C., Dehm S.M., Sharifi N. (2023). Targeting the Androgen Signaling Axis in Prostate Cancer. J. Clin. Oncol..

[25] Cheng B., Huang H. (2023). Expanding horizons in overcoming therapeutic resistance in castration-resistant prostate cancer: Targeting the androgen receptor-regulated tumor immune microenvironment. Cancer Biol. Med..

[26] Mattes M.D. (2024). Overview of Radiation Therapy in the Management of Localized and Metastatic Prostate Cancer. Curr. Urol. Rep..

[27] (2023). Metastasis-directed therapy plus hormone therapy improves progression-free survival for advanced prostate cancer. Cancer.

[28] Sindhu K.K., Nehlsen A.D., Stock R.G. (2022). Radium-223 for Metastatic Castrate-Resistant Prostate Cancer. Pract. Radiat. Oncol..

[29] Sorensen B.S., Horsman M.R. (2020). Tumor Hypoxia: Impact on Radiation Therapy and Molecular Pathways. Front. Oncol..

[30] Cheng G., Zielonka J., Ouari O., Lopez M., McAllister D., Boyle K., Barrios C.S., Weber J.J., Johnson B.D., Hardy M. (2016). Mitochondria-Targeted Analogues of Metformin Exhibit Enhanced Antiproliferative and Radiosensitizing Effects in Pancreatic Cancer Cells. Cancer Res..

[31] D’Hose D., Mathieu B., Mignion L., Hardy M., Ouari O., Jordan B.F., Sonveaux P., Gallez B. (2022). EPR Investigations to Study the Impact of Mito-Metformin on the Mitochondrial Function of Prostate Cancer Cells. Molecules.

[32] Benej M., Hong X., Vibhute S., Scott S., Wu J., Graves E., Le Q.T., Koong A.C., Giaccia A.J., Yu B. (2018). Papaverine and its derivatives radiosensitize solid tumors by inhibiting mitochondrial metabolism. Proc. Natl. Acad. Sci. USA.

[33] Oshima N., Ishida R., Kishimoto S., Beebe K., Brender J.R., Yamamoto K., Urban D., Rai G., Johnson M.S., Benavides G. (2020). Dynamic Imaging of LDH Inhibition in Tumors Reveals Rapid In Vivo Metabolic Rewiring and Vulnerability to Combination Therapy. Cell Rep..

[34] Ashton T.M., Fokas E., Kunz-Schughart L.A., Folkes L.K., Anbalagan S., Huether M., Kelly C.J., Pirovano G., Buffa F.M., Hammond E.M. (2016). The anti-malarial atovaquone increases radiosensitivity by alleviating tumour hypoxia. Nat. Commun..

[35] Skwarski M., McGowan D.R., Belcher E., Di Chiara F., Stavroulias D., McCole M., Derham J.L., Chu K.Y., Teoh E., Chauhan J. (2021). Mitochondrial Inhibitor Atovaquone Increases Tumor Oxygenation and Inhibits Hypoxic Gene Expression in Patients with Non-Small Cell Lung Cancer. Clin. Cancer Res..

[36] Beerkens A.P.M., Boreel D.F., Nathan J.A., Neuzil J., Cheng G., Kalyanaraman B., Hardy M., Adema G.J., Heskamp S., Span P.N. (2024). Characterizing OXPHOS inhibitor-mediated alleviation of hypoxia using high-throughput live cell-imaging. Cancer Metab..

[37] Nenu I., Gafencu G.A., Popescu T., Kacso G. (2017). Lactate—A new frontier in the immunology and therapy of prostate cancer. J. Cancer Res. Ther..

[38] Wang J.X., Choi S.Y.C., Niu X., Kang N., Xue H., Killam J., Wang Y. (2020). Lactic Acid and an Acidic Tumor Microenvironment suppress Anticancer Immunity. Int. J. Mol. Sci..

[39] Alcorn S., Walker A.J., Gandhi N., Narang A., Wild A.T., Hales R.K., Herman J.M., Song D.Y., Deweese T.L., Antonarakis E.S. (2013). Molecularly targeted agents as radiosensitizers in cancer therapy—Focus on prostate cancer. Int. J. Mol. Sci..

[40] Angel M., Zarba M., Sade J.P. (2021). PARP inhibitors as a radiosensitizer: A future promising approach in prostate cancer?. Ecancermedicalscience.

[41] Ahn H.K., Lee Y.H., Koo K.C. (2020). Current Status and Application of Metformin for Prostate Cancer: A Comprehensive Review. Int. J. Mol. Sci..

[42] Lord S.R., Harris A.L. (2023). Is it still worth pursuing the repurposing of metformin as a cancer therapeutic?. Br. J. Cancer.

[43] Hua Y., Zheng Y., Yao Y., Jia R., Ge S., Zhuang A. (2023). Metformin and cancer hallmarks: Shedding new lights on therapeutic repurposing. J. Transl. Med..

[44] Gupta P.K., Orlovskiy S., Arias-Mendoza F., Nelson D.S., Nath K. (2024). (1)H and (31)P Magnetic Resonance Spectroscopic Metabolomic Imaging: Assessing Mitogen-Activated Protein Kinase Inhibition in Melanoma. Cells.

[45] Gupta P.K., Orlovskiy S., Arias-Mendoza F., Nelson D.S., Osborne A., Pickup S., Glickson J.D., Nath K. (2024). Metabolic Imaging Biomarkers of Response to Signaling Inhibition Therapy in Melanoma. Cancers.

[46] Nath K., Nelson D.S., Heitjan D.F., Leeper D.B., Zhou R., Glickson J.D. (2015). Lonidamine induces intracellular tumor acidification and ATP depletion in breast, prostate and ovarian cancer xenografts and potentiates response to doxorubicin. NMR Biomed..

[47] Nath K., Nelson D.S., Heitjan D.F., Zhou R., Leeper D.B., Glickson J.D. (2015). Effects of hyperglycemia on lonidamine-induced acidification and de-energization of human melanoma xenografts and sensitization to melphalan. NMR Biomed..

[48] Nath K., Nelson D.S., Ho A.M., Lee S.C., Darpolor M.M., Pickup S., Zhou R., Heitjan D.F., Leeper D.B., Glickson J.D. (2013). ^31^P and ^1^H MRS of DB-1 melanoma xenografts: Lonidamine selectively decreases tumor intracellular pH and energy status and sensitizes tumors to melphalan. NMR Biomed..

